# Evaluating the Immunogenic Potential of ApxI and ApxII from *Actinobacillus pleuropneumoniae*: An Immunoinformatics-Driven Study on mRNA Candidates

**DOI:** 10.3390/vetsci12050414

**Published:** 2025-04-27

**Authors:** Yi Deng, Jia-Yong Chen, Yuhan Wang, Yu-Luo Wang, Jiale Liu, Zhiling Peng, Jiayu Zhou, Kun Lu, Xin Wen, Xizhu Chen, Siyu Pang, Dan Wang, Miaohan Li, Senyan Du, San-Jie Cao, Qin Zhao

**Affiliations:** 1 Research Center for Swine Diseases, College of Veterinary Medicine, Sichuan Agricultural University, Chengdu 611130, China; dengyi@stu.sicau.edu.cn (Y.D.); chenjiayong@stu.sicau.edu.cn (J.-Y.C.); wangyuhan1@stu.sicau.edu.cn (Y.W.); 2024303131@stu.sicau.edu.cn (Y.-L.W.); liujiale@stu.sicau.edu.cn (J.L.); 202001774@stu.sicau.edu.cn (Z.P.); 202200986@stu.sicau.edu.cn (J.Z.); lukun@stu.sicau.edu.cn (K.L.); 2024203038@stu.sicau.edu.cn (X.W.); 2024303120@stu.sicau.edu.cn (X.C.); 202000826@stu.sicau.edu.cn (S.P.); 2024203037@stu.sicau.edu.cn (D.W.); limiaohan@stu.sicau.edu.cn (M.L.); senyandu@sicau.edu.cn (S.D.); 2 Sichuan Science-Observation Experimental Station of Veterinary Drugs and Veterinary Biotechnology, Ministry of Agriculture and Rural Affairs, Chengdu 611130, China; 3 International Joint Research Center of Animal Disease Control and Prevention, Science & Technology Department of Sichuan Province, Chengdu 611130, China; 4 Key Laboratory of Animal Disease and Human Health of Sichuan Province, Science & Technology Department of Sichuan Province, Chengdu 611130, China

**Keywords:** *Actinobacillus pleuropneumoniae*, immunoinformatics, potential candidate antigens, cross-protection, mRNA vaccine

## Abstract

Porcine infectious pleuropneumonia (PCP) caused by *Actinobacillus pleuropneumoniae* (APP) leads to severe economic losses in swine production. Commercial vaccines offer limited cross-protection for the 19 serotypes, while APP mRNA vaccines remain unexplored. This study evaluated eight candidate APP proteins (ApxI-IV, OlmA, TbpB, GalT, and GalU) using immunobioinformatics tools, and their immunogenicity and cross-protection were assessed in a mouse model. The results revealed that ApxI and ApxII excel due to their stability, strong antigenicity, non-sensitization, and high immune receptor affinity. Compared to the PBS group, both ApxI and ApxII induced higher serum IgG, IL-2, IL-4, and IFN-γ levels. Following challenge with the two most prevalent APP strains in Mainland China, APP 5b and APP 1, the survival rates for ApxI (71.4% and 62.5%) and ApxII (75% and 71.4%) were measured, with notably reduced lung lesions and neutrophil infiltration. These findings highlight ApxI and ApxII’s potential in mRNA vaccine development as a promising approach to overcome current vaccine limitations. Future research should focus on creating APP mRNA vaccines and testing their efficacy in swine. This study is the first to combine immunoinformatics with experimental validation for APP mRNA vaccine antigens, representing a novel contribution.

## Simple Summary

Porcine infectious pleuropneumonia (PCP), a highly contagious and pathogenic respiratory disease, presents significant challenges in vaccine development. This study employed immunoinformatics to assess eight candidate antigens from *Actinobacillus pleuropneumoniae* (APP). Within a murine model, ApxI and ApxII were identified as the most promising candidates, demonstrating stability, high antigenicity, lack of sensitization, and strong affinity for immune receptors. These antigens elicited robust IgG responses and conferred protection against strains of APP serotypes 1 and 5b. These findings underscore the potential of ApxI and ApxII as mRNA antigen candidates for APP, laying the groundwork for future swine trials to overcome current vaccine limitations. As an innovative investigation in mRNA vaccine technology, this study established a novel methodological framework that integrates theoretical calculations with experimental validation by combining immunoinformatics prediction with in vitro assessments of APP antigens, making significant original contributions to the field.

## 1. Introduction

Porcine infectious pleuropneumonia (PCP) is a highly contagious and fatal infectious disease in swine, caused by *Actinobacillus pleuropneumoniae* (APP) and characterized by hemorrhagic fibrinous pneumonia and fibrinous necrotizing pleurisy [1,2]. According to differing capsule and lipopolysaccharide antigenicity, 19 serotypes of APP have been reported [3]. APP strains are categorized based on their nicotinamide adenine dinucleotide (NAD) requirements during in vitro culture into NAD-dependent biotype I (serotypes 1–12, 15, and 16) and NAD-independent biotype II (serotypes 13 and 14) [4]. Biotype II strains generally cause milder infections than biotype I strains [5]. Within biotype I, serotypes 1, 5, 9, and 11 are highly virulent, often leading to severe outbreaks, lung disease, and increased mortality. Serotypes 1 and 5 are further divided into subtypes 1a and 1b and 5a and 5b, with notable differences in virulence; subtype 1a is typically more toxic, grows faster in liquid culture, and exhibits higher pathogenicity in animal studies [6]. The 5b subtype is more prevalent in Europe and may be more virulent than 5a due to its unique genomic traits [1]. APP has 19 serotypes, each dominant in different regions [7]. In China, serotypes 1, 3, 5, and 7 are common [4], while North America frequently reports serotypes 3, 5, 6, 7, 8, 15, and 17 [2,8]. In Europe, serotypes 2, 3, 8, and 13 are prevalent [2,9]. Virulence can vary by region, for instance, European serotype 2 strains are highly toxic, whereas North American strains are not [10].

APP strains with different serotypes can produce four types of cell toxins, named ApxI, ApxII, ApxIII, and ApxIV, belonging to the repeats-in-toxin (RTX) family. Research has shown that Apx toxins play an essential role in the pathogenicity of APP, especially ApxI, which has strong hemolytic and cytotoxic characteristics. It plays a significant role in hemolysis and cell damage [11,12,13], leading to severe lung lesions [14]. The ApxII toxin has the characteristics of low hemolysis and moderate cytotoxicity [1]. The toxic effect of the ApxII toxin mainly causes severe inflammation and necrosis of the lungs by dissolving host cells, manifested as typical lung lesions. In the acute phase, it leads to fibrinous hemorrhagic necrotizing pneumonia and vascular thrombosis. In the chronic phase, it may cause pleural adhesions and abscesses. Its virulence is enhanced with other toxins, such as ApxI [15,16]. The ApxIII toxin has a strong toxic effect on monocytes in peripheral blood. Compared with other Apx toxins, the ApxIII toxin does not have hemolytic activity. Still, its high cytotoxicity causes it to play an essential role in the infection process, manifested as typical severe inflammation and necrosis of the lungs [1]. ApxIV is a toxin specific to APP, expressed exclusively in vivo across all serotypes, and serves as a critical target for diagnostic and vaccine development efforts [17]. Recent research highlights the synergistic action of various RTX toxins in APP, indicating that the finely tuned regulation of ApxIV expression in terms of timing and spatial distribution may enhance the bacterium’s adaptability. This regulatory mechanism facilitates its effective colonization and integration into pre-existing microbial communities on the airway mucosal surface [18]. Outer membrane protein (OMP) is also an important virulence factor of APP. OMP plays an essential role in maintaining cellular integrity and serum resistance, as well as in the biofilm formation of APP, and it has been confirmed that it can help APP escape through the body’s barrier in immune evasion [19]. Besides the Apx toxins and OMP, other virulence factors of APP, such as TbpB, GalT, and GalU, contribute to its pathogenicity. TbpB is a transferrin-binding protein involved in iron acquisition, whereas the OlmA protein is a member of the OMP family, both proteins are significant constituents of vaccine antigens [20]. GalT and GalU are implicated in synthesizing bacterial polysaccharides, influencing bacterial survival and virulence [21]. It has been confirmed that innovative immuno-prophylactic preparations of APP based on ApxI, ApxII, ApxIII, and ApxIV toxins and outer membrane proteins (OMPs), among others (i.e., RnhB, GalU, GalT, HflX, ComL, LolB, LppC, TbpB, and OlmA), have high protective efficacy in mice and pigs [22,23,24]. In short, APP toxins are crucial for their pathogenicity and serve as key targets for vaccine development. Understanding their mechanisms and immunogenicity can guide the creation of effective vaccines and treatments.

PCP is widely prevalent in most pig-raising countries worldwide, causing substantial economic losses to the pig industry [4]. The economic repercussions of APP are predominantly attributed to the high morbidity and mortality rates observed in infected swine, which lead to diminished productivity and elevated expenditures related to veterinary care and disease management. The pathogen’s propensity for swiftly developing antimicrobial resistance further exacerbates treatment challenges, imposing additional economic strains on swine producers [25,26]. Beyond the direct economic losses stemming from reduced productivity and increased veterinary expenses, APP infections can precipitate secondary economic effects. For instance, severe pleurisy and pneumonia lesions induced by APP can diminish average daily gain and carcass weight in finishing pigs, resulting in heightened production costs and reduced profitability. The mean daily profit is projected to decline by 34%, whereas the feed conversion ratio is anticipated to decrease by 26% [27]. Economic modeling indicates that pigs with elevated pleurisy scores incur additional costs per kilogram and experience reduced total revenue and return on investment [28]. Pigs recovered from acute infection and chronically infected pigs can be carrier animals that shed the pathogen continuously without clinical symptoms. Thus, it is difficult to control and eradicate this disease in infected herds [29].

The inactivated whole-cell vaccine is the most commonly utilized commercial vaccine for preventing PCP in pig farms. However, its efficacy is limited, providing only partial protection against the disease. They offer limited cross-protection against different serotypes and may cause side effects such as inflammation, fever, and behavioral changes in pigs, impacting their growth and incurring economic losses [30,31]. Additionally, these vaccines may not induce as strong an immune response as subunit or combination vaccines [32]. Thus, exploring better vaccine strategies to enhance protection against PCP is essential. Traditional vaccine development methodologies have predominantly relied on in vivo or in vitro techniques, which are characteristically time-consuming, labor-intensive, and costly [33]. However, recent advancements in computational biology and immunoinformatics have facilitated the emergence of alternative vaccine design strategies, notably in silico methods [34]. These computational approaches utilize computer-based tools and algorithms to analyze biological data and predict immunogenic peptides that may serve as potential vaccine candidates [35]. Importantly, tools such as T/B cell epitope prediction (IEDB), signal peptide prediction (Signal 6.0 Server), SOPMA (self-optimized prediction method with alignment), SWISS-MODEL, ABCpred, and molecular docking platforms (HADDOCK, Cluspro, and HDOCK) have been successfully applied in the development of mRNA vaccines. The IEDB Analysis Resource is crucial in developing mRNA vaccines by facilitating bioinformatics analysis and design. It was pivotal in the creation of a multi-epitope mRNA vaccine for Streptococcus agalactiae, where it predicted CTL, HTL, and B cell epitopes to construct a vaccine capable of eliciting both humoral and cell-mediated immune responses, thereby highlighting the significance of epitope prediction in vaccine design [36]. The Signal 6.0 Server enhances mRNA vaccine efficacy by optimizing signal sequences to improve antigen expression and immune response, primarily through increased translation efficiency. This is corroborated by simulations demonstrating enhanced SRP54 binding and translation efficiency. This optimization in the context of SARS-CoV-2 mRNA vaccines resulted in elevated antigen levels and enhanced immune protection [37]. SOPMA is a predictive tool for protein secondary structures in mRNA vaccine design, plays a significant role in this process by aiding in the identification of these epitopes. By analyzing the secondary structure of proteins, SOPMA helps researchers predict regions that are likely to be antigenic, thus facilitating the design of multi-epitope vaccines [38]. SWISS-MODEL is instrumental in predicting the three-dimensional structures of proteins, a critical component in designing mRNA vaccines to optimize sequences for enhanced immune response and stability. By analyzing antigen structures, SWISS-MODEL informs the design process, and research indicates that computational tools can effectively redesign mRNA to improve vaccine efficacy and accelerate development [39]. ClusPro contributes to mRNA vaccine design by predicting protein interactions with immune receptors, such as toll-like receptors (TLRs), as evidenced by a study on a human parechovirus vaccine utilizing TLR4 [40]. In the context of mRNA vaccine design, HADDOCK facilitates the simulation of docking interactions between vaccine antigens and TLRs, which are essential for initiating the innate immune response. For instance, in the development of a multi-epitope mRNA vaccine targeting the Hendra virus, strong binding affinities with TLR-4 and TLR-2 were observed, with interaction scores of −1139.4 KJ/mol and −1277.9 KJ/mol, respectively, indicating stable interactions crucial for eliciting a robust immune response [41]. In summary, computational modeling tools have been pivotal in designing and developing mRNA vaccines, significantly enhancing the capacity to address the challenges of infectious diseases rapidly.

In this article, we aim to explore the potential of immunoinformatics approaches for candidate antigens against APP, including ApxI, ApxII, ApxIII, ApxIV, TbpB, OlmA, GalT, and GalU. At the same time, the immunogenicity of the dominant antigen proteins ApxI and ApxII in the murine model was evaluated by experimental study. We will discuss the underlying principles of immunoinformatics and highlight the computational tools and algorithms commonly employed in identifying and characterizing vaccine candidates. By leveraging these computational strategies, we anticipate that the comparative analysis of candidate proteins and the development of an mRNA vaccine targeting APP can be expedited, ultimately contributing to improved prevention and control of APP-associated diseases.

## 2. Materials and Methods

### 2.1. Obtaining Protein Sequences

In this study, the protein sequences were retrieved from the National Center for Biotechnology Information (NCBI) [42] (accessible at https://www.ncbi.nlm.nih.gov/, accessed on 30 September 2023) and were (1) ApxI (accession number AAK50051.1), (2) ApxII (accession number AAK50052.1), (3) ApxIII (accession number AAK50053.1), (4) ApxIV (accession number AAD01698.1), (5) TbpB (accession number AAA21928.1), (6) OlmA (accession number AAC41456.1), (7) GalT (accession number WP_005601381.1), and (8) GalU (accession number WP_005600805.1), which were identified as potential protective antigens [22].

### 2.2. Fundamental Characterization

The physicochemical properties of the proteins were analyzed using the online software ProtParam (accessible at http://web.expasy.org/protparam/, accessed on 30 November 2023) [43], including the number of amino acids, the molecular weight, the theoretical pI, the instability index, the aliphatic index, and the grand average of hydropathicity (GRAVY). The online website GnegmPLoc (accessible at http://www.csbio.sjtu.edu.cn/bioinf/Gneg-multi/, accessed on 3 December 2023) [44] was used to predict the subcellular localization of the eight proteins. AlgPred 2.0 (accessible at https://webs.iiitd.edu.in/raghava/algpred2/batch.html, accessed on 3 December 2023) was used to indicate the allergenicity of bacterial protein sequences by using a hybrid (RF + BLAST + MERCI) approach, with a selection threshold of 0.3 [45]. This was performed in order to conduct a more comprehensive analysis of the selected proteins.

### 2.3. Prediction of Antigenicity

Given the critical role of antigenicity in vaccine development, we employed the Immune Epitope Database (IEDB) website (accessible at https://www.iedb.org/, accessed on 6 December 2023) to predict the antigenicity of these eight proteins. Based on the studies conducted by Talukdar et al. [46] and Lata et al. [47], a threshold antigen score exceeding 1.0 suggests that the corresponding protein exhibits high antigenicity for antigenicity scoring.

### 2.4. Prediction of Signal Peptides

To assess the presence of signal peptides in the selected protein, we utilized SignalP 6.0 (accessible at https://services.healthtech.dtu.dk/services/SignalP-6.0/, accessed on 4 December 2023) [48]. This comprehensive analysis was performed on the protein sequence using SignalP 6.0, a deep learning-based tool capable of predicting the presence or absence of signal peptides across various domains, including archaea, Gram-positive bacteria, Gram-negative bacteria, and eukaryotic proteins. Additionally, it identifies cleavage sites where signal peptides are present. The prediction results are accompanied by clear probability scores, offering a quantitative foundation for identifying signal peptides. To enhance the effectiveness of cell epitopes, we selected a sequence devoid of signal peptides [49].

### 2.5. Prediction of Secondary Structure

The secondary structures of the eight selected proteins were predicted utilizing the SOPMA server (accessible at https://npsa-prabi.ibcp.fr/NPSA/npsa_sopma.html, accessed on 30 November 2023), employing the default parameter settings.

### 2.6. Prediction of Tertiary Structure

The tertiary structure of the protein determines its biological function. This paper used SWISS-MODEL [35] (accessible at https://swissmodel.expasy.org/, accessed on 30 November 2023) to predict the tertiary structure of the 8 proteins. The optimal configuration of the tertiary structure was selected according to GMQE, Seq Identity, and Coverage.

### 2.7. Prediction of Linear B Cell Epitopes

The linear B cell epitopes were predicted using the online server ABCpred [50] (accessible at https://webs.iiitd.edu.in/raghava/abcpred/ABC_submission.html, accessed on 11 December 2023). ABCpred predicts linear B cell epitopes using an artificial neural network (ANN). Each chosen protein was submitted individually with a threshold of 0.51. The length of epitopes was selected as 16mer, and the overlap filter remained active [49]. The top five epitopes of the results were studied further.

### 2.8. Prediction of T Cell Epitopes 

Cytotoxic T cell lymphocyte (CTL) epitopes are linear peptides that bind and present SLA-I molecules after antigen processing by antigen-presenting cells. With high specificity, CTL epitopes play a key role in antigen recognition, presentation, and cellular immunity. Cytotoxic T cell lymphocyte (CTL) epitopes were predicted using the NetMHCpan 4.1 EL method through the Immune Epitope Database MHC I, which can be accessed at IEDB [51] (accessible at http://tools.iedb.org/main/tcell/, accessed on 6 December 2023). NetMHCpan 4.1 EL utilized the eluted ligand (EL) score and classified results based on percentile rank. In vaccine development, the pig MHC I allele SLA-1*0401 is chosen for its high expression and widespread presence in pigs, enhancing vaccine effectiveness and coverage. Its diversity and high-frequency expression across breeds make it an ideal vaccine target [52], aiding T cell epitope prediction and broad immune response design [53].

### 2.9. Molecular Docking

The three-dimensional (3D) structures of the select proteins were modeled using SWISS-MODEL, and the Protein Data Bank (PDB) identifiers for MHC I alleles (SLA-1) (PDB ID: O19244), MHC II alleles (SLA-DRB1) (PDB ID: O77499), and toll-like receptor 2 (TLR-2) (PDB ID: Q59HI8) were sourced from the UniProt Knowledge Base (UniProtKB). Molecular docking simulations were performed utilizing the ClusPro server (accessible at https://cluspro.org/login.php, accessed on 10 December 2023) [54] the HDOCK server (accessible at http://hdock.phys.hust.edu.cn/, accessed on 24 December 2023) [55], and the HADDOCK server (accessible at https://rascar.science.uu.nl/haddock2.4/, accessed on 5 December 2023) [56]. The top-ranked interaction results, determined according to the scoring criteria of each server, were selected for further analysis and comparison. Full-length protein sequences were employed for the molecular docking simulations with relevant immune receptor molecules.

### 2.10. Bacterial Strains, Media, and Culture Conditions

Our lab isolated and identified the two most common APP strains in Mainland China: APP 1 (serotype 1, strain 4074, GenBank: CP030753.1) and APP 5b (serotype 5b, strain L20, GenBank: CP000569.1). The LD50 in BALB/c mice was 5.0 × 10^6^ CFU for APP 1 and 5.0 × 10^7^ CFU for APP 5b. Both strains were cultured in TSB or TSA with 10% calf serum and 10 μg/mL NAD at 37 °C, 220 rpm. E. coli DH5α was used for cloning, and E. coli BL21 for protein expression, both cultured in LB medium with kanamycin at 37 °C, 220 rpm.

### 2.11. Cloning and Expression of the Dominant Antigenic Proteins ApxI and ApxII from APP

The predominant antigen proteins underwent codon optimization and were subsequently synthesized by GenScript Biotech (Nanjing, China) for mRNA vaccine development. The proteins were then cloned into a pET28a plasmid using BamHI and XhoI restriction sites, incorporating an N/C-terminal 6x His-tag. The primer sequences are provided in Table 1. The optimized sequences and sequencing results are presented in Figures S1 and S2. The constructs were transformed into *E. coli* BL21 (DE3) for recombinant expression. Induction with 1 mM isopropyl-beta-D-1-thiogalactopyranoside (IPTG) (Solarbio, Beijing, China) was performed for 5 h once the optical density (OD) of the bacterial culture reached 0.6 to 0.8 at 37 °C.

### 2.12. Preparation and Purification of the Predominant Antigen Proteins ApxI and ApxII

#### 2.12.1. Bacterial Lysis

For this, 200 mL of cell culture was centrifuged at 4500× *g* for 20 min to collect the cell pellets. The supernatant was discarded and 20 mL of precooled cell lysis buffer was added, composed of 50 mM Tris-HCl (pH 8.0), 5 mM EDTA, 100 mM NaCl, 0.1% NaN3, 0.5% Triton X-100, 0.1 mM PMSF, and 1 mM DTT, to initiate cell lysis.

#### 2.12.2. Protein Ultrasonication

The resuspended cells were subjected to ultrasonication using an ultrasonic disruptor set at 180 W, applying 3 s ultrasound pulses followed by 2 s intervals for 30 min while maintaining the sample on ice. Subsequently, the lysate was centrifuged at 6000× *g* for 15 min at 4 °C. The sonication and centrifugation process was repeated twice on the resultant precipitate. After the final centrifugation, the modified lysate, devoid of Triton X-100, was employed for terminal fragmentation.

#### 2.12.3. Protein Purification

The purification of proteins was conducted following previously established protocols [57]. Protein precipitates were isolated via centrifugation at 5000× *g* for 30 min and resuspended in 1 mL of dissolving buffer comprising 50 mM Tris-HCl (pH 8.0) and 100 mM NaCl. To this solution, 3 mL of 8 M urea was added to attain a final urea concentration of 2 M. The inclusion body solution was then dissolved on ice and centrifuged at 5000× *g* for 10 min to collect the supernatant. Proteins were sequentially dissolved and purified at urea concentrations of 2 M, 4 M, 6 M, and 8 M, with centrifugation performed after each step. All samples were stored at −80 °C.

### 2.13. SDS-PAGE and Determination of Protein Concentration

SDS-PAGE analysis assessed the purity of the isolated recombinant proteins ApxI and ApxII. A 10% polyacrylamide gel was prepared for SDS-PAGE to identify recombinant ApxI and ApxII proteins, running at 80 V for 20 min and 120 V for 60 min. The gel was stained with Coomassie Blue Fast Staining Solution (Tiangen, Beijing, China) for 30 min at room temperature and decolorized by boiling until a transparent background was achieved. The expected molecular weights of the ApxI and ApxII proteins are 110 kDa and 105 kDa, respectively.

An ultra-micro nucleic acid protein analyzer (Jiapeng, Shanghai, China) was used to measure the protein concentration.

### 2.14. Western Blotting

The initial step was performed as described for SDS-PAGE, followed by the transfer of proteins onto polyvinylidene difluoride (PVDF) membranes. A mouse anti-His-Tag monoclonal antibody (Abmart, Shanghai, China) was used as the primary antibody at a dilution of 1:5000. The secondary antibody was a horseradish peroxidase (HRP)-conjugated goat anti-mouse IgG (Boster, Wuhan, China), also diluted at 1:5000. Visualization of the membranes was achieved using Clarity Western ECL Substrate (BIO-RAD, Hercules, CA 94547, USA). Images were captured with a Chemi Doc imaging system (BIO-RAD, Hercules, CA 94547, USA).

### 2.15. Animals

Forty-five five-week-old female specific pathogen-free (SPF) BALB/c mice were purchased from Sibeifu (Beijing, China) Biotechnology Co., Ltd. (Beijing, China) under animal production license no. SCXK (Jing) 2024-0001. The animal experiment protocol for this study received approval from the Experimental Animal Ethics and Welfare Committee of Sichuan Agricultural University (approval number: 20240132). All mice were housed under standard laboratory conditions, with a controlled temperature range of 22 to 26 °C, relative humidity of 50% to 60%, and a 12 h light/dark cycle. The mice had ad libitum access to chow and water.

### 2.16. Vaccination and Challenge of BALB/c Mice 

To assess the immunogenicity of ApxI and ApxII, mice were immunized according to the established protocol outlined in Table 2, utilizing a subcutaneous route. A group injected with PBS served as the negative control. The adjuvant employed was 10% MONTANIDE^TM^ Gel 02 ST. Immunizations were administered on days 0 and 14, with no mortality observed throughout the study. Serum samples were collected from each mouse via orbital vein bleeding on days 0, 7, and 21 to monitor the immune response. A cytokine assay was conducted on the serum of each mouse on day 21. The results of the tests are presented as means ± standard deviation (SD). Two weeks after the booster immunization, all groups of mice were challenged intraperitoneally with a lethal dose of 2 × 10^8^ colony-forming units (CFU) of APP 5b and 3.0 × 10^7^ CFU of APP 1. The mice were carefully monitored daily for seven days post-challenge to assess respiratory symptoms, general illness, and mortality. Lung tissues were collected for histopathologic analysis and immunohistochemical analysis. Mortality data were recorded throughout the seven-day post-challenge period.

### 2.17. Determination of IgG Levels

An indirect enzyme-linked immunosorbent assay (ELISA) was employed to quantify the levels of specific IgG antibodies generated by immunized mice. Purified ApxI and ApxII proteins were diluted to a concentration of 1 µg/100 µL using a carbonate buffer at pH 9.6. Subsequently, 100 µL of the diluted protein solution was dispensed into each well of a 96-well plate, followed by incubation at 4 °C for 10–12 h. After incubation, the coating solution was removed, and the wells were washed three times with 200 µL of washing buffer, each wash lasting 5 min. A blocking step was performed by adding 100 µL of 5% nonfat bovine serum albumin, incubating at 37 °C for 1.5 h. The wells were then washed three times, each for 5 min. Serum samples were serially diluted in a 2-fold gradient with 5% skim milk in PBST and added to the antigen-coated wells (100 µL per well) in triplicate for each dilution. Negative controls were included, and the plates were incubated at 37 °C for 1 h. Following incubation, the wells were washed three times for 5 min each. A horseradish peroxidase-conjugated goat anti-mouse IgG (H + L) antibody, diluted at 1:5000, was added at 100 µL per well, and the plates were incubated at room temperature for 1 h. The plates underwent a washing procedure consisting of three cycles, each lasting five minutes. Subsequently, 100 µL of soluble TMB substrate solution was introduced into each well, followed by a 15 min incubation period at room temperature under dark conditions. The enzymatic reaction was halted by adding 100 µL of ELISA stop solution to each well. The absorbance at 450 nm was measured using a microplate reader (BIO-RAD, Hercules, CA 94547, USA) on a 96-well plate.

### 2.18. Detection of Cytokine Expression Levels

IL-2, IL-4, and INF-γ assays were performed on the serum of immunized mice on day 21 using mouse ELISA kits (ABclonal, Wuhan, China) according to the manufacturer’s specifications. Each mouse represents an independent biological replicate sample and cytokines were expressed in pg/mL.

### 2.19. HE Staining and Histopathological Analysis

The lungs were fixed in 4% paraformaldehyde, resulting in well-preserved specimens. The lung tissues underwent trimming, dehydration, embedding, sectioning, and staining with hematoxylin-eosin. They were then sealed and subjected to microscopic examination to ensure specimen quality. Histological sections, with a thickness of 3 µm, were analyzed using an optical Eclipse Ci microscope (Nikon, Tokyo, Japan). Section scanning utilized CaseViewer2.4 (3DHISTECH, Budapest, Hungary). A blinded methodology was consistently employed for both image acquisition and analysis.

### 2.20. Immunohistochemistry

Lung tissue underwent a series of preparatory procedures, including pruning, dehydration, embedding, and sectioning, resulting in histological sections with a thickness of 3 µm. Neutrophils were stained by the specific Anti-Myeloperoxidase (MPO) Mouse mAb (Servicebio, Wuhan, China) to assess neutrophil infiltration in the lungs of mice. After staining, the sections were analyzed using an Eclipse Ci optical microscope (Nikon, Tokyo, Japan) at 400× magnification. Images were captured with standardized exposure settings and analyzed using ImageJ2 software (Version 2.16.0; NIH, Bethesda, MD, USA). The intensity of the positive signal was quantified by measuring the integrated optical density (IOD), thereby assessing the extent of neutrophil infiltration in the lung tissue. To ensure objectivity, a blind method was consistently employed throughout the entire image acquisition and analysis process.

### 2.21. Statistical Analyses

All statistical analyses were conducted using SPSS 27.0 software, while Graphpad Prism 9.1 was utilized for data visualization. One-way analysis of variance (ANOVA) was employed to determine significant differences among the groups. A *p*-value of <0.05 was considered statistically significant and denoted by an asterisk (*). *p*-values of <0.01 and <0.001 are indicated by two (**) and three (***) asterisks, respectively.

## 3. Results

### 3.1. Physicochemical Properties of Proteins

The protein sequences of the eight proteins obtained from NCBI will be included in the Supplementary Information. The online analysis software ProtParam analyzed the amino acid sequences of eight potential candidate antigen proteins of APP, and the instability index of seven proteins—ApxI, ApxII, ApxIII, ApxIV, TbpB, OmlA, and GalU—were all <40, indicating that they are all stable proteins. The instability index of the GalT protein was 45.96, which means that it is unstable and easy to degrade. Moreover, the aliphatic index of the eight proteins was less than 100, and the grand average of hydropathicity (GRAVY) was negative, meaning that they are all hydrophilic proteins. Additionally, the AlgPred server indicated that all of the other proteins were likely non-allergenic, except for the GalU protein (Table 3).

### 3.2. The Antigenicity of Proteins

The antigenicity of the eight selected proteins was assessed using the IEDB online software. Among these proteins, TbpB exhibited an antigenicity value of 0.999, which is notably close to 1.0. The antigenicity values of the remaining seven proteins exceeded 1.0, specifically ApxI (1.015), ApxII (1.013), ApxIII (1.012), ApxIV (1.000), OlmA (1.003), GalT (1.025), and GalU (1.038), indicating strong antigenic potential (Supplementary Figure S3).

### 3.3. Signal Peptides of Proteins

According to the predicted results of SignalP 6.0, proteins ApxI, ApxII, ApxIII, ApxIV, GalT, and GalU had no signal peptide. In contrast, TbpB and OmlA had lipoprotein signal peptides (Supplementary Figure S4). Signal peptides primarily function to direct proteins through the secretion pathway, culminating in either their extracellular secretion or localization to the cell membrane. These peptides’ structural and functional attributes significantly influence proteins’ biological activity, expression efficiency, and immunogenicity [58]. Research indicates that post-translational modifications (PTMs) within the eukaryotic secretion pathway can attenuate the immune response elicited by nucleic acid vaccines incorporating natural prokaryotic signal peptide proteins [59]. Based on the predicted results, the proteins ApxI, ApxIII, ApxIV, GalT, and GalU, which lack signal peptides, exhibit significant potential for developing APP mRNA vaccines.

### 3.4. The Secondary Structure of Proteins

According to the predicted results of SOPMA, the proportions of four different secondary structures of the eight proteins, namely the alpha helix (Hh), extended strand (Ee), beta turn (Tt), and random coil (Cc), are shown (Supplementary Table S1). Among the secondary structures of the eight proteins, ApxII, ApxIII, ApxI, and GalU were dominated by alpha helices, with values above 40% for all four. Among them, the alpha helix structure of ApxII was the highest (52%), followed by ApxIII (48.19%), ApxI (46%), and GalU (41.02%). The second most common structure of these four proteins was random coils, in the following order: GalU (36.27%), ApxII (26.26%), ApxIII (25.48%), and ApxI (25.44%). The other proteins were mainly made up of random coils, including TbpB (58.52%), OlmA (57.81%), GalT (49.28%), and ApxIV (38.50%). In addition, the second most common structure of TbpB (24.11%) and OlmA (20.00%) was extended strands. For GalT (29.51%), the second most common structure was the alpha helix, while the alpha helix (26.37%) and the extended strand (26.15%) were evenly distributed in ApxIV.

### 3.5. The Tertiary Structure of Proteins

According to the SWISS-MODEL predictive analysis, the model with the highest GMQE, Seq Identity, and Coverage values was selected as the tertiary structure of the selected protein (Supplementary Figure S5). According to the predicted results, the tertiary structure conformations of all eight proteins corresponded to their secondary structural features.

### 3.6. Prediction and Estimation of B Cell Epitopes

In this study, epitope selection was restricted to the top five predictions for each protein, as determined by the ABCpred webserver (Table 4). According to the prediction analysis, the top five B cell epitopes of all eight proteins had high prediction scores (>0.8) [60].

### 3.7. Prediction and Estimation of the CTL Epitopes

The researchers used the IEDB database to identify the potential CTL epitopes from the eight proteins studied in this study. Each protein was ranked according to its percentile rank, and the top five epitopes of each protein are presented in Table 5. The analysis revealed that the calculated percentile ranks of the top five epitopes for the ApxI, ApxII, ApxIV, and TbpB proteins were below 0.2, indicating a high binding affinity with MHC-I molecules [61]. For the OlmA, GalT, and GalU proteins, only the calculated percentile ranks of their first two epitopes were below 0.2, which suggests a high affinity. Based on the analysis of SCORE and RANK, the epitopes with the highest affinity for each of the eight proteins are identified as follows: ApxI (KTDTGYLTF), ApxII (FTDRGIVLF), ApxIII (KIDEWEKKY), ApxIV (LLDPNSYYY), TbpB (KAEKATTSY), OlmA (KIDNFPLKL), GalT (TVADNKPSY), and GalU (IADKPLIQY).

### 3.8. Molecular Dynamics Simulation

According to the scoring criteria of the three molecular docking servers, the most stable docking model was selected for analysis. Lower model binding values indicated higher binding affinity and more stable binding. For each model, the most stable complex model was chosen for comparison (Table 6). In summary, all eight proteins could form relatively stable molecular docking complexes with MHC I alleles (SLA-1), MHC II alleles (SLA-DRB1), and toll-like receptor 2 (TLR-2). The scoring values of the ApxI, ApxII, and ApxIV protein docking complexes were high and consistent in the three servers.

### 3.9. Cloning, Expression, and Identification of Inclusion Body Proteins

**Figure 1:**
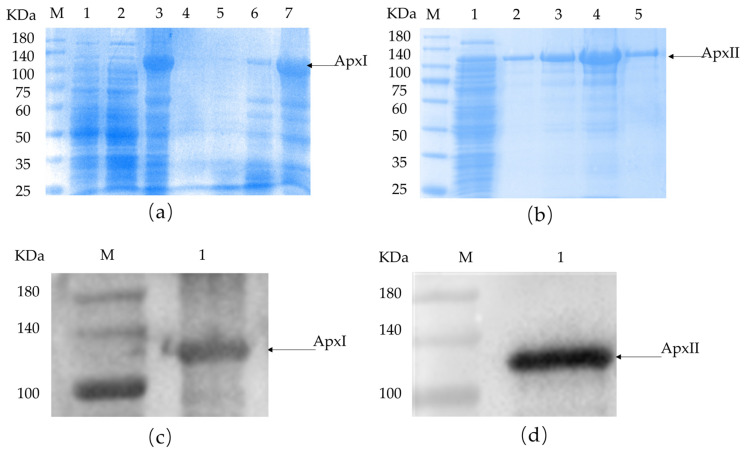
SDS-PAGE and Western blotting identification of ApxI and ApxII. (**a**) SDS-PAGE identification of ApxI. M: Maker; 1: before induction; 2: supernatant; 3: inclusion bodies; 4–8: ApxI in different urea concentrations. (**b**) SDS-PAGE identification of ApxII. M: Maker; 1: inclusion bodies; 2–5: ApxII in different urea concentrations. (**c**) Western blotting identification of ApxI. M: Maker; 1: The target band of ApxI. (**d**) Western blotting identification of ApxII. M: Maker; 1: The target band of ApxII.

According to bioinformatics analysis, proteins with good antigenicity, a stable structure, no signal peptide, and high molecular docking stability were selected for cloning and expression. ApxI and ApxII were chosen to be cloned as a full-length protein. The recombinant plasmids were successfully transformed into *E. coli* BL21 and the respective proteins were expressed as His-tagged fusion proteins. Both ApxI and ApxII were expressed in inclusion bodies (Figure 1), then lysed and sonicated in denaturing conditions. SDS-PAGE and Western blotting examined the proteins on 10% gels based on the predicted molecular weight of each protein. SDS-PAGE confirmed that these fusion proteins were expressed in *E. coli* BL21 with the expected molecular mass (Figure 1). ApxI was described correctly and had the highest solubility at 8 M urea with few miscellaneous proteins (Figure 1a). ApxII was expressed correctly and dissolved in all urea concentrations. The highest solubility was observed at 6 M urea, but miscellaneous proteins were also increased, while a higher purity of the protein was obtained at urea concentrations of 2 M and 8 M (Figure 1b). At the same time, there was a corresponding exposure stripe on the Western blotting results. The size of the target band of ApxI was about 110 kDa and the target band of ApxII had a size of about 105 kDa, which were consistent with the expected band sizes, indicating that ApxI and ApxII were successfully expressed (Figure 1c,d).

The concentration of the recombinant ApxI protein, as measured by a micro protein nucleic acid analyzer, was 3.54 mg/mL, whereas the concentration of the ApxII protein was 2.424 mg/mL.

### 3.10. Antibody Level Assays

**Figure 2:**
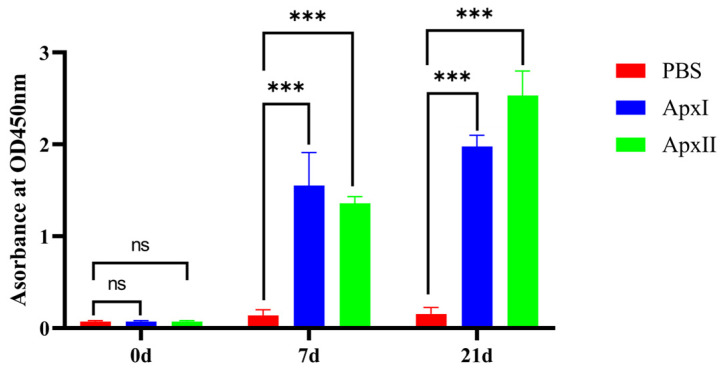
Analysis of levels of IgG. Serum samples were collected before and 1 week after each immunization. Antibody levels were tested using an indirect ELISA coated with the corresponding recombinant proteins. Antibody levels were demonstrated as absorbance at 450 nm. Data are represented as means ± SD. n = 3. The ns indicates no significant difference from the control group (*p* > 0.05), *** *p* < 0.001.

Serum samples were collected before and 1 week after each immunization (0 d, 7 d, and 21 d). An indirect ELISA was used to test the IgG levels. Referring to the results of the indirect ELISA (Figure 2), the IgG levels of every group were increased by relatively large margins compared with the serum before immunization. Compared with the PBS group, the IgG levels of the ApxI and ApxII antigen protein groups were significantly elevated (*p* < 0.001). The results showed that ApxI and ApxII could generate a robust humoral immune response.

### 3.11. Cytokine Level Assays

**Figure 3:**
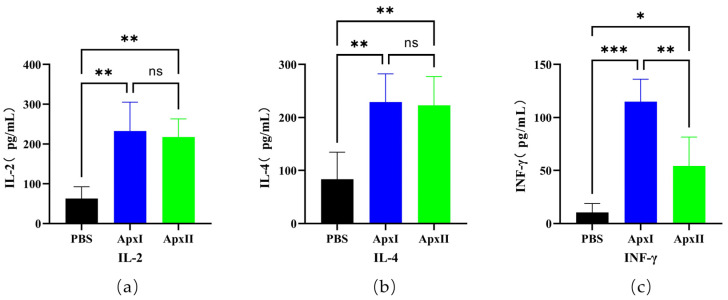
The cytokines IL-2, IL-4, and IFN-γ in the serum of mice. (**a**) IL-2 levels in the serum of immune ApxI and ApxII. (**b**) IL-4 levels in the serum of immune ApxI and ApxII. (**c**) IFN-γ levels in the serum of immune ApxI and ApxII. Data are represented as means ± SD. n = 5. The ns indicates no significant difference from the control group (*p* >0.05), * *p* < 0.05, ** *p* < 0.01, *** *p* < 0.001.

Immune serum samples from the PBS, ApxI, and ApxII groups were diluted 1:4 times on day 21, and the cytokines IL-2, IL-4, and IFN-γ were detected using a double-antibody sandwich ELISA (Figure 3). The results showed that the expression levels of IL-2 (*p* < 0.01), IL-4 (*p* < 0.01), and IFN-γ (*p* < 0.001) in the serum of mice in the ApxI group were significantly higher than those in the PBS group. The expression levels of IL-2 (*p* < 0.01), IL-4 (*p* < 0.01), and IFN-γ (*p* < 0.05) in the serum of mice in the ApxII group were also significantly increased. This suggests that ApxI and ApxII effectively increased humoral and cellular immune responses.

### 3.12. Protective Efficiency in a Murine Model

**Figure 4:**
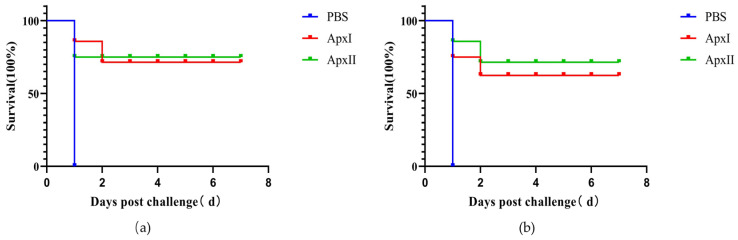
Protective efficiency of ApxI and ApxII in a murine model. (**a**) Survival rate of mice challenged with APP 5b after immunization. (**b**) Survival rate of mice challenged with APP 1 after vaccination (n = 7).

The vaccination and challenge experiments results showed that after 12 h of challenge, some mice showed clinical symptoms such as listlessness, huddling, shortness of breath, closed eyes, and ruffled hair. All mice in the PBS group died within 24 h after challenge. After each immunization group was challenged with a lethal dose of APP 5b, ApxI and ApxII provided 71.4% and 75.0% protection, respectively (Figure 4a). After each immunization group was challenged with a lethal dose of APP 1, ApxI and ApxII provided 62.5% and 71.4% protection, respectively (Figure 4b). This indicated that ApxI and ApxII could offer better protection against APP 5b and APP 1 infections (Figure 4).

### 3.13. Hematoxylin-Eosin (HE) Staining

**Figure 5:**
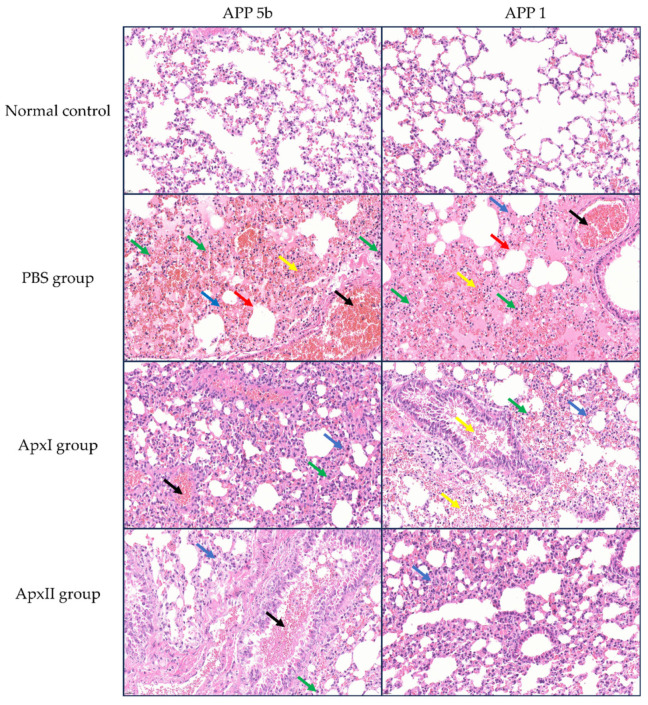
HE staining analysis of lung tissues from mice in each immunization group post-challenge with APP 5b and APP 1 (magnification 400×). Black arrows indicate vascular congestion; yellow arrows denote alveolar hemorrhage; green arrows highlight inflammatory cell infiltration; blue arrows point to alveolar septal thickening; and red arrows mark epithelial cell necrosis (n = 3).

The HE staining examination of lung tissues is shown in Figure 5. Following the challenge with APP 5b and APP 1 strains, mice in the PBS group exhibited significant lung tissue damage, characterized by alveolar hemorrhage, vascular congestion, thickening of the alveolar septa, necrosis of epithelial cells, and infiltration of inflammatory cells. Conversely, the pathological damage to lung tissues in the groups immunized with ApxI and ApxII was mitigated to varying extents. The administration of ApxI and ApxII effectively reduced lung damage in mice challenged with APP 5b and APP 1 strains, thereby providing protective effects.

### 3.14. Immunohistochemical Analysis

**Figure 6:**
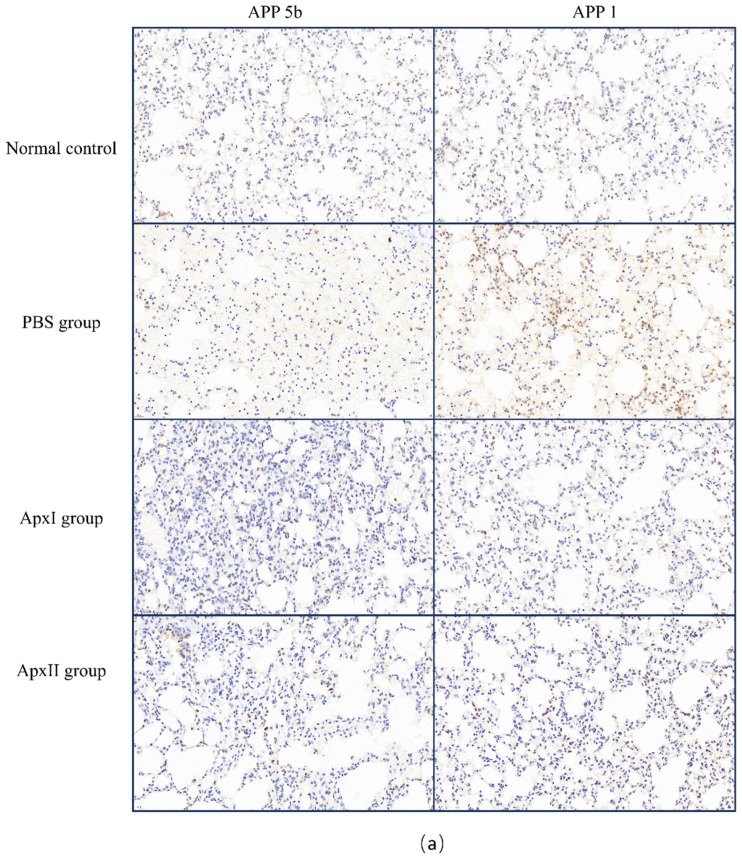

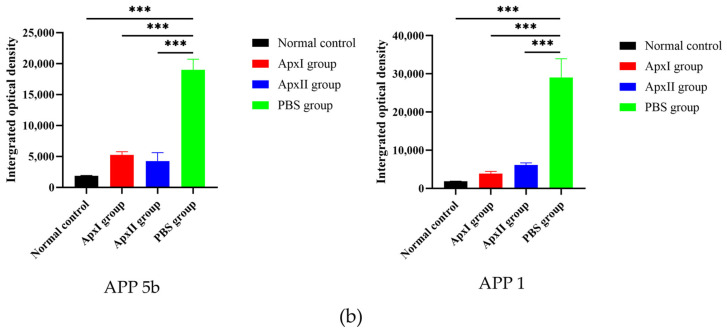
Immunohistochemical analysis of neutrophil infiltration in the pulmonary tissue of mice across different immunization groups. (**a**) Neutrophil infiltration in the lung tissue of mice within each immunization group, examined at 400× magnification. (**b**) The IOD values of lung neutrophils for each group were analyzed using ImageJ2 software (Version 2.16.0; NIH, Bethesda, MD, USA). The normal group was used as the negative control. Data are represented as means ± SD. n = 3. *** *p* < 0.001.

Following the APP 5b and APP 1 challenges, neutrophil infiltration in the mice from each immunization group was assessed using immunohistochemistry (Figure 6). The PBS group’s IOD values were 1.9 × 10^4^ for APP 5b and 2.9 × 10^4^ for APP 1, indicating substantial neutrophil infiltration. In contrast, the IOD values for the ApxI group were 5.2×10^3^ for APP 5b and 3.9 × 10^3^ for APP 1, while the ApxII group exhibited IOD values of 4.3 × 10^3^ for APP 5b and 6.1 × 10^3^ for APP 1. Compared to the PBS group, there was a significant reduction in neutrophil infiltration within the lung tissue of the ApxI and ApxII groups. These findings suggest that ApxI and ApxII confer adequate protection against lung inflammation in mice.

## 4. Discussion

PCP is attributed to APP, a respiratory pathogen that results in considerable health detriments within swine production systems [28]. Since the disease’s initial identification, extensive research has been conducted globally, leading to the continuous development and enhancement of various APP vaccines with protective efficacy. The conventional approach to controlling PCP largely relies on inactivated whole-cell APP vaccines. However, these traditional vaccines do not confer cross-protection [30]. Research has identified several key virulence factors and antigens of APP, such as Apx toxins, OMP, transferrin-binding proteins (Tbp) [62], and in vivo induced antigens [23], as potential candidates for vaccine development. Nonetheless, the possible application of these antigens in mRNA vaccine formulations remains unexplored. mRNA vaccines have emerged as a formidable alternative to traditional vaccines due to their high potency, safety, efficacy, rapid clinical development capabilities, and potential for cost-effective and swift production [63]. Some studies suggest bacterial mRNA vaccines offer robust and superior protection [59,64]. The data presented highlight potential pathways for developing effective antibacterial vaccines that are urgently needed. Despite this, there is no documentation on creating an mRNA vaccine.

Vaccination remains one of the most promising strategies for the treatment of PCP. Nevertheless, the design and development of effective vaccines necessitate substantial investments and resources. The emergence and re-emergence of infectious diseases (ERID), contagious agents with complex lifecycle and antigenic variability, and the requirement for personalized vaccination further complicate vaccine development [65]. Consequently, only a limited number of vaccine candidates successfully reach the market. Traditional methods are both time-intensive and costly [66]. However, recent advancements in computational biology and immunoinformatics offer alternative strategies for vaccine design. Various computational tools and software have been developed to expedite vaccine design and development [67]. This research methodology has been extensively applied in the research and development of mRNA vaccines, including those targeting bacteria [68], viruses [69], and cancers [70].

This study represents a pioneering effort to integrate immune informatics with the experimental validation of APP antigens within the framework of mRNA vaccine development, highlighting significant innovation. Initially, the potential of eight candidate antigen proteins from APP (ApxI, ApxII, ApxIII, ApxIV, OlmA, TbpB, GalT, and GalU) as mRNA vaccines was evaluated using immunoinformatics techniques. The physicochemical properties of these candidate proteins were analyzed through online platforms, focusing on attributes such as molecular weight, theoretical isoelectric point (pI), and hydropathicity, which are critical for determining the stability and immunogenicity of the vaccine [33]. Subcellular localization was assessed to ascertain the protein’s cellular destinations, while allergenicity was evaluated to ensure vaccine safety. The antigenic score, indicative of the proteins’ capacity to elicit an immune response, was notably high. Apart from TbpB, which scored 0.999 (approaching 1.0), the other seven antigens demonstrated antigenicity scores exceeding 1.0, suggesting robust antigenic potential. All proteins except for GalT were found to be stable, and all except for GalU were determined to be non-allergenic. ApxI, ApxII, ApxIII, ApxIV, OlmA, and TbpB demonstrated favorable physicochemical properties and antigenicity, suggesting their potential as viable vaccine candidates. Previous studies have primarily focused on the immunoinformatics analysis of specific characteristics of one or a limited number of antigens of APP. In contrast, this study provides a comprehensive analysis of eight candidate antigens of APP, offering a more systematic and thorough examination that enhances and supplements existing information on APP antigens.

Additionally, Signal 6.0 was employed to predict the signal peptide of the proteins. Removing signal peptides can optimize protein expression and functionality in the context of vaccine design and protein expression, ensuring their activity in the appropriate environment. Consequently, OlmA and TbpB are deemed unsuitable for direct use in vaccine design. The secondary and tertiary structures of the proteins were predicted using SOPMA and SWISS-MODEL, respectively. This structural information is crucial for understanding the interactions between the vaccine and the immune system [71]. Additionally, discontinuous B cell epitopes were identified using the ABCpred webserver, and CTL epitopes were identified using the IEDB database, which provided insights into the spatial arrangement of antigenic regions [72]. Identifying B cell epitopes in antigens is crucial in developing recombinant vaccines or immunotherapies for various diseases [73]. Therefore, it was analyzed in this study and can be used to develop an APP epitope mRNA vaccine in the follow-up study. In addition, identifying T cell epitopes is somewhat complex and challenging [74], and further analysis is needed, but this information is equally helpful for subsequent studies.

The 3D structure of the selected proteins and MHC I alleles (SLA-1), MHC II alleles (SLA-DRB1), and toll-like receptor 2 (TLR-2) were docked using the ClusPro server, HDOCK server, and HADDOCK server. A major histocompatibility complex (MHC), including MHC I and MHC II, is present on the cell surface and can recognize and bind antigens from the outside world [75,76]. TLRs are crucial in recognizing pathogen-associated molecular patterns and initiating immune responses [77]. As a membrane surface receptor, TLR2 is the most promiscuous TLR for recognizing pathogen-associated molecular patterns derived from bacteria, viruses, parasites, and fungi, and its activation can result in the functioning of the intracellular signaling pathway of nuclear factor-kappa B and cytokine production, leading to innate immunity activation [78]. The ClusPro server computed the models based on electrostatic interactions and desolvation energy [79], and a model complex with the lowest binding energy score was chosen. The HDOCK website utilizes a semi-flexible docking approach while considering non-bonding interactions, including electrostatic interactions and van der Waals forces, to forecast its binding mode and affinity. The website provided a list of the top ten docking models, and we selected the first model for evaluation [49]. Each HADDOCK simulation generates 200 models for the TLR2/protein complex, ranked, scored, and clustered based on structural similarity. The most reliable cluster has the lowest HADDOCK score and Z-score [80]. According to the comprehensive comparative analysis of the results of three docking websites, among the eight proteins, ApxI, ApxII, and ApxIV could form stable complexes with all the ligands and had stable and good binding affinities, which were critical for the initiation of innate immune responses against foreign antigens. They act as pattern recognition receptors, allowing the immune system to detect invading microorganisms and pathogens and mount an appropriate defense. Previously, there have been reports of epitope vaccine research using bioinformatics methods to analyze single or multiple APP proteins. Still, they are not comprehensive and are mainly used for epitope screening [81]. In this paper, we used this method to analyze, compare, and screen eight potential candidate antigen proteins of APP and predicted the binding affinity of these candidate antigens with immune molecules. This not only enriches and complements the study of APP antigen proteins but also provides a basis for developing APP mRNA vaccines. In recent years, bioinformatics and computational technology advancements have significantly enhanced the role of immune informatics and computational tools in antigen screening for bacterial mRNA vaccines. In alignment with our research strategy, Bernhardt et al. [82] employed bioinformatics tools to identify potential vaccine candidate peptides for *Staphylococcus aureus* that are capable of binding to MHC class I molecules and activating CD8+ T cells, thereby serving as promising vaccine components. Similarly, Alhassan [83] employed computational tools such as IEDB, ProtParam, and the ClusPro 2.0 molecular docking platform to design multi-epitope vaccines targeting *Escherichia coli.* Furthermore, Ullah et al. [84] developed a multi-epitope mRNA vaccine using tools such as IEDB and ABCpred for the study of the *Rhipicephalus microplus* tick, delivering it via lipid nanoparticles (LNPs). This approach not only enhances the vaccine’s stability but also its immunogenicity. Consequently, the vaccine design strategy proposed in this study not only addresses the current target pathogen, APP, but also possesses the potential for broader application to other pathogens, including bacteria and parasites, due to its modular strategy and computationally driven approach.

Based on the results of bioinformatics analysis, ApxI and ApxII were selected for experimental verification to study their immune properties. We performed the vaccine trial using ApxI and ApxII. This research demonstrated that the ApxI and ApxII groups exhibited significantly higher antibody response levels compared to the PBS group. These findings align with those reported by Byung-Sun Park, further corroborating the immunogenic potential of ApxI and ApxII [85]. The cytokines IL-2 and IFN-γ are known to activate Th1 responses, while IL-4 is associated with the stimulation of Th2 responses. IFN-γ, secreted by NK cells and T cells, plays a pivotal role in the immune response [86]. The induction of Th1 or Th2 immune responses can lead to either humoral or cellular immunity [87]. In our study, the increased levels of IL-2, IL-4, and IFN-γ observed in animals vaccinated with ApxI or ApxII proteins further confirmed the robust immunological potential of these proteins. Additionally, ApxI and ApxII conferred cross-protection in a mouse challenge model, including protection against APP serotypes 5b and 1. The ability to provide better cross-protection than whole-cell bacterins confirmed the potential of ApxI and ApxII for the development of APP mRNA vaccines [22]. In addition, the pathological analysis of lung tissue showed that compared with the PBS group, the lung lesions in the ApxI and ApxII groups were significantly reduced, and the lesions in the ApxII group were milder than those in the ApxI group, indicating that ApxII may have a better protective effect on lung tissue. In the immunohistochemical assay, the inflammatory response was significantly reduced in both the ApxI and ApxII groups. This is consistent with other findings showing that subunit vaccines effectively reduce bacterial load and alleviate tissue damage and lesions caused by bacterial infection [88]. Although Figure 6 compares neutrophil chemotaxis across experimental groups by excluding necro-hemorrhagic foci, we recognize the spatial heterogeneity of the inflammatory response in lungs infected with APP. The periphery of necrotic lesions, which represents the active interface between bacterial toxins and host immune responses, shows the highest intensity of neutrophil infiltration. Excluding these regions may lead to an underestimation of the overall chemotactic activity. This situation parallels diagnostic challenges in clinical pathology, where distinguishing between primary neutrophilic infiltration and secondary inflammatory responses remains a subject of debate. Future research employing spatial transcriptomics or multiplex immunohistochemistry could provide a more comprehensive understanding of these dynamics. The results from the vaccination and mice challenge experiments demonstrated that both ApxI and ApxII possess immunoprotective properties against APP serotypes 5b and 1. Collectively, these findings propose that ApxI and ApxII are promising candidate antigens for the development of an APP mRNA vaccine. Nevertheless, this study is subject to several limitations. These include the exclusive use of mouse models rather than the native porcine model, the absence of actual mRNA construct development, and the lack of comparative analysis of immune protection effects with commercially available subunit vaccines, inactivated vaccines, and gene-deficient live vaccines as control groups. Consequently, future research will prioritize the development of mRNA vaccines targeting ApxI and ApxII for PCP, emphasizing the evaluation of their immunoprotective effects in porcine models.

## 5. Conclusions

This study underscores the significant role of immune informatics methodologies in designing vaccines against APP, offering a more rapid and precise strategy for identifying and selecting optimal candidate antigens. By employing a suite of computational tools and algorithms, the candidate antigen proteins ApxI and ApxII were identified as the most promising based on a comprehensive analysis of their physical and chemical properties, antigenicity, binding epitopes, allergenicity, and ligand binding affinity among eight potential antigen proteins. Subsequently, recombinant ApxI and ApxII were utilized for immunization in murine models, with APP 1 and APP 5b strains used for challenge immunization. The protective efficacy of ApxI against APP 1 and APP 5b was determined to be 62.5% and 71.4%, respectively, while ApxII conferred protection rates of 71.4% and 75% against the same strains. Post-immunization with these proteins resulted in a marked reduction in pulmonary inflammation, thereby conclusively demonstrating the efficacy of ApxI and ApxII in mitigating APP-related challenges in mice. In conclusion, this study represents a pioneering effort to integrate immunoinformatics with the experimental validation of APP antigens within the framework of mRNA vaccine development. It constitutes a novel and groundbreaking contribution to the field. This work establishes a foundational reference for the future development of mRNA vaccines targeting PCP.

## Figures and Tables

**Table 1:** The sequences of the ApxI and ApxII amplification primers.

Primer Direction	Sequence (5′–3′)	Product (bp)
ApxI-F	CagcaaatgggtcgcggatccATGGCCAATTCACAACTGGACA ^1^	3069
ApxI-R	gtggtggtggtggtgctcgagGGCGACTGCGTTAAGTAATTG	
ApxII-F	cagcaaatgggtcgcggatccATGTCCAAAATAACCC	2871
ApxII-R	gtggtggtggtggtgctcgagGGCGGCCCTGGCCAGCTG	

^1^ The sites for restriction enzymes are highlighted with an underline. Lowercase letters denote the homology arm of the homologous recombination junction, while uppercase letters represent the sequence amplification primer for the protein.

**Table 2:** The immunization and challenge procedures in mice.

Group	Boost Immunization Dose (Day 0)	Secondary Immunization Dose (Day 14)	Challenge Dose of APP 5b (Day 28)	Challenge Dose of APP 1 (Day 28)
PBS (n = 14)	200 μL (n = 14)	200 μL (n = 14)	2 × 10^8^ CFU (n = 7)	2 × 10^8^ CFU (n = 7)
ApxI (n = 14)	160 μg/200 μL (n = 14)	80 μg/200 μL (n = 14)	2 × 10^8^ CFU (n = 7)	2 × 10^8^ CFU (n = 7)
ApxII (n = 14)	160 μg/200 μL (n = 14)	80 μg/200 μL (n = 14)	2 × 10^8^ CFU (n = 7)	2 × 10^8^ CFU (n = 7)
Normal (n = 3)	/ ^1^	/	/	/

^1^ The slash indicates no treatment and was used as normal control mice.

**Table 3:** List of the basic structural and physicochemical properties of amino acids.

Features	ApxI	ApxII	ApxIII	ApxIV	TbpB	OlmA	GalT	GalU
Number of amino acids	1022	956	1052	1805	593	365	349	364
Molecular weight	110,439.71	102,495.27	112,862.99	202,130.45	65,528.9	39,783.91	40,574.76	40,526.7
Theoretical pI	5.5	5.88	5.62	4.81	6.6	5.74	6.06	5.78
Instability index	25.74	25.58	18.6	20.42	26.85	24	45.96	35.94
Aliphatic index	88.63	91.2	87.46	78.02	65.62	74	69.89	89.73
Grand average of hydropathicity (GRAVY)	−0.352	−0.365	−0.37	−0.61	−0.683	−0.679	−0.64	−0.125
Subcellular localization	Extracell	Extracell	Extracell	Extracell	Cell inner/outer membrane	Cell outer membrane	Cell inner membrane	Cytoplasm
Allergenicity	Non-Allergen	Non-Allergen	Non-Allergen	Non-Allergen	Non-Allergen	Non-Allergen	Non-Allergen	Allergen

**Table 4:** The list of the top five B cell epitopes.

Protein Name	Rank	Sequence	Start	Score
ApxI	1	RGIALFAPQFDKLLNK	102	0.96
	2	KLLEKDPDRFDKKVFD	507	0.95
	3	GGDGNDHLVGGNGNDR	766	0.92
	3	PDLSLAGPGFDAVSGI	299	0.92
	4	GGTLYYHEDYNGNALT	893	0.91
ApxII	1	GGAGDDVIDGGNGNNF	791	0.94
	1	HETIATHPTNVGNREE	711	0.94
	2	TTVIDGGDGHDRVHYS	663	0.93
	3	GNDGDDHLFGGAGDDV	782	0.92
	3	ADFHRETGTIDASVTT	387	0.92
ApxIII	1	GNDGDDILYGDKGNDE	800	0.94
	2	AGKGDDKLYGSSGSDL	864	0.92
	2	GGEGNDKLLGGNGNNY	827	0.92
	2	GDKGNDELRGDNGNDQ	809	0.92
	3	GSSGSDLLDGGEGNDY	873	0.91
ApxIV	1	AFYIERKNGGGAKNNS	606	0.97
	2	HTAYEERNDFLAGNNI	813	0.95
	2	LESIIDPSGIGGTVNL	283	0.95
	2	KGHGQDIVYEDTNNDN	1730	0.95
	3	TVESTDSSASVVRVTP	938	0.94
TbpB	1	TETGEKRNERVVELSE	132	0.97
	2	EREIDGFDTSGDGKNV	257	0.95
	2	FKQGIDGYVYYLGVTP	217	0.95
	3	GKVISYKGTWDFVSNI	239	0.94
	4	AHSSEFAVDFDNKKLT	300	0.93
OlmA	1	DGTIINGTLYSKIDNF	345	0.97
	2	GGSSGSSSKPNSELTP	64	0.95
	3	TKSISYKGDMFYSYKD	274	0.92
	4	EPKNMAPQMGNPKLND	121	0.92
	5	NEMNIAESERVARMNI	29	0.89
GalT	1	LFNISFPYSMGFHFAP	330	0.92
	1	VVPYWAGWPFETLLLP	282	0.92
	1	SALLPDTPAPEAGSDP	140	0.92
	2	MVGYEMMAESQRDLTP	374	0.91
	2	YSMGFHFAPFNESDNP	337	0.91
GalU	1	HSQIMVAPVPREDVSS	216	0.92
	2	EAFHMTGRTFDCGDKL	309	0.90
	3	HATERIDYLYLTRANS	25	0.87
	4	PGLCSEPHSPHATERI	15	0.85
	5	QAFTEYSLRHDKFGND	328	0.83

**Table 5:** The list of the top five CTL epitopes.

Protein Name	Allele	Start	Peptide	Score	Rank
ApxI	SLA-1*0401	641	KTDTGYLTF	0.993859	0.01
		311	ALAISPLSF	0.846586	0.09
		579	GVQSHNAIY	0.825595	0.11
		331	QLEQYSERF	0.821156	0.11
		99	FSERGIALF	0.797568	0.14
ApxII	SLA-1*0401	103	FTDRGIVLF	0.962448	0.02
		552	RIQEGKNSY	0.928137	0.03
		86	NLDIAKTSF	0.915631	0.04
		311	ALAVSPLSF	0.860726	0.08
		895	ALSKVGNDY	0.81843	0.12
ApxIII	SLA-1*0401	435	KIDEWEKKY	0.989124	0.01
		566	RTQTGKYEY	0.869036	0.07
		865	STSGNHTIY	0.825199	0.11
		322	SLAISPLAF	0.824904	0.11
		1014	KIISSANTF	0.815365	0.12
ApxIV	SLA-1*0401	541	LLDPNSYYY	0.994525	0.01
		879	ATDLQYQHY	0.990917	0.01
		960	ITDKVNNMY	0.987556	0.01
		1577	RVDNDLMLF	0.986716	0.01
		356	KLDNPLAPY	0.982951	0.01
TbpB	SLA-1*0401	39	KAEKATTSY	0.96172	0.02
		390	KIDLAGVNF	0.961356	0.02
		62	KLMEPALGY	0.950251	0.02
		4	KLNPYALAF	0.890933	0.05
		326	KAEEMAGKF	0.773911	0.17
OlmA	SLA-1*0401	315	KIDNFPLKL	0.936361	0.03
		272	KVFGENNDY	0.816159	0.12
		236	ISYKGDMFY	0.677654	0.31
		305	GTIINGTLY	0.639769	0.37
		311	TLYSKIDNF	0.63462	0.39
GalT	SLA-1*0401	41	TVADNKPSY	0.91327	0.04
		138	QANELKTRY	0.762714	0.18
		260	ALKKLTTRY	0.730635	0.21
		62	ITGEQNPVY	0.685695	0.29
		289	ESDNPHWQL	0.645757	0.35
GalU	SLA-1*0401	28	IADKPLIQY	0.993868	0.01
		198	SVEEAPSNL	0.791608	0.15
		88	SIVPKDVTL	0.458822	0.75
		256	RTFDCGDKL	0.439677	0.81
		89	IVPKDVTLM	0.437322	0.82

**Table 6:** Molecular docking of the 8 proteins.

Protein Name	ClusPro Weighted Score			HADDOCK Z-Score			HDOCK Z-Score		
	SLA-1	SLA-DRB1	TLR2	SLA-1	SLA-DRB1	TLR2	SLA-1	SLA-DRB1	TLR2
ApxI	−1475.9	−1468.4	−1767.0	−116.5	−114.5	−117.3	−270.40	−289.00	−284.27
ApxII	−1223.1	−1239.0	−1312.5	−101.4	−113.5	−113.5	−259.37	−304.47	−312.96
ApxIII	−1315.6	−1263.4	−1441.7	−139.7	−119.5	−66.2	−274.27	−343.92	−304.81
ApxIV	−1376.5	−1283.8	−1596.4	−112,3	−121.5	−123.3	−288.24	−278.83	−300.93
TbpB	−1331.3	−1358.4	−1323.1	−67.8	−56.7	−104.7	−261.82	−239.04	−253.36
OlmA	/ ^1^	−1200.3	/	−29.6	−65.4	−99.7	−229.41	−266.37	−245.48
GalT	−1139.6	−990.6	−1308.9	−66.1	−116.4	−70.8	−282.58	−309.45	−286.46
GalU	−1080.9	−1083.5	−1358.9	−29.5	−65.3	−39.0	−243.57	−258.07	−235.93

^1^ The slash indicates no docking model.

## Data Availability

All data analyzed during this study are available from the corresponding author upon reasonable request.

## Supplementary Materials

The following supporting information can be downloaded at: https://www.mdpi.com/article/10.3390/vetsci12050414/s1, Figures S1 and S2. Comparative analysis of ApxI and ApxII original sequence, optimized sequence, and sequencing sequence. Figure S3. The antigenicity prediction results of the selected 8 proteins. Figure S4. The prediction results of signal peptides for the selected 8 proteins. Figure S5. The prediction results of the tertiary structures of the selected 8 proteins. Figures S6–S9. Original SDS-PAGE and Western Blotting images of ApxI and ApxII. Table S1. The list of secondary structure predictions of the selected 8 proteins.

## Abbreviations

The following abbreviations are used in this manuscript:

PCP	Porcine contagious pleuropneumonia
APP	*Actinobacillus pleuropneumoniae*
galT	galactose-1-phosphate uridyltransferase
GalU	UTP-glucose-1-phosphate uridylyltransferase
IPTG	Isopropyl-beta-D-1-thiogalactopyranoside
TLR-2	Toll-like receptor 2
