# Supplementary material for: Evaluating the Immunogenic Potential of ApxI and ApxII from Actinobacillus pleuropneumoniae: An Immunoinformatics-Driven Study on mRNA Candidates
Source: Vet Sci. 2025 Apr 27;12(5):414. doi: 10.3390/vetsci12050414 (PMC12115502; doi:10.3390/vetsci12050414)
Supplement: Supplementary file 1 [file vetsci-12-00414-s001.zip › Supplementary File/Table S1.The list of secondary structure predictions of the selected 8 proteins.pdf]

**Table S1.** The list of secondary structure predictions of the selected 8 proteins.

| | **Hh**[1] | **Ee**[2] | **Tt**[3] | **Cc**[4] |
|---|---|---|---|---|
| ApxI | 47.46% | 18.59% | 8.51% | 25.44% |
| ApxII | 52.00% | 15.9% | 5.75% | 26.26% |
| ApxIII | 48.19% | 17.40% | 8.49% | 25.48% |
| ApxIV | 26.37% | 26.15% | 8.98% | 38.50% |
| TbpB | 11.30% | 24.11% | 6.07% | 58.52% |
| OlmA | 18.17% | 20.00% | 4.38* | 57.81% |
| GalT | 29.51% | 15.16% | 5.44% | 49.28% |
| GalU | 41.02% | 17.63% | 5.08% | 36.27% |

[1]Hh： Alpha helix; [2]Ee： Extended strand; [3]Tt: Beta turn; [4]Cc: Random coil.
