# Supplementary figures and images for: Evaluating the Immunogenic Potential of ApxI and ApxII from Actinobacillus pleuropneumoniae: An Immunoinformatics-Driven Study on mRNA Candidates
Source: Vet Sci. 2025 Apr 27;12(5):414. doi: 10.3390/vetsci12050414 (PMC12115502; doi:10.3390/vetsci12050414)

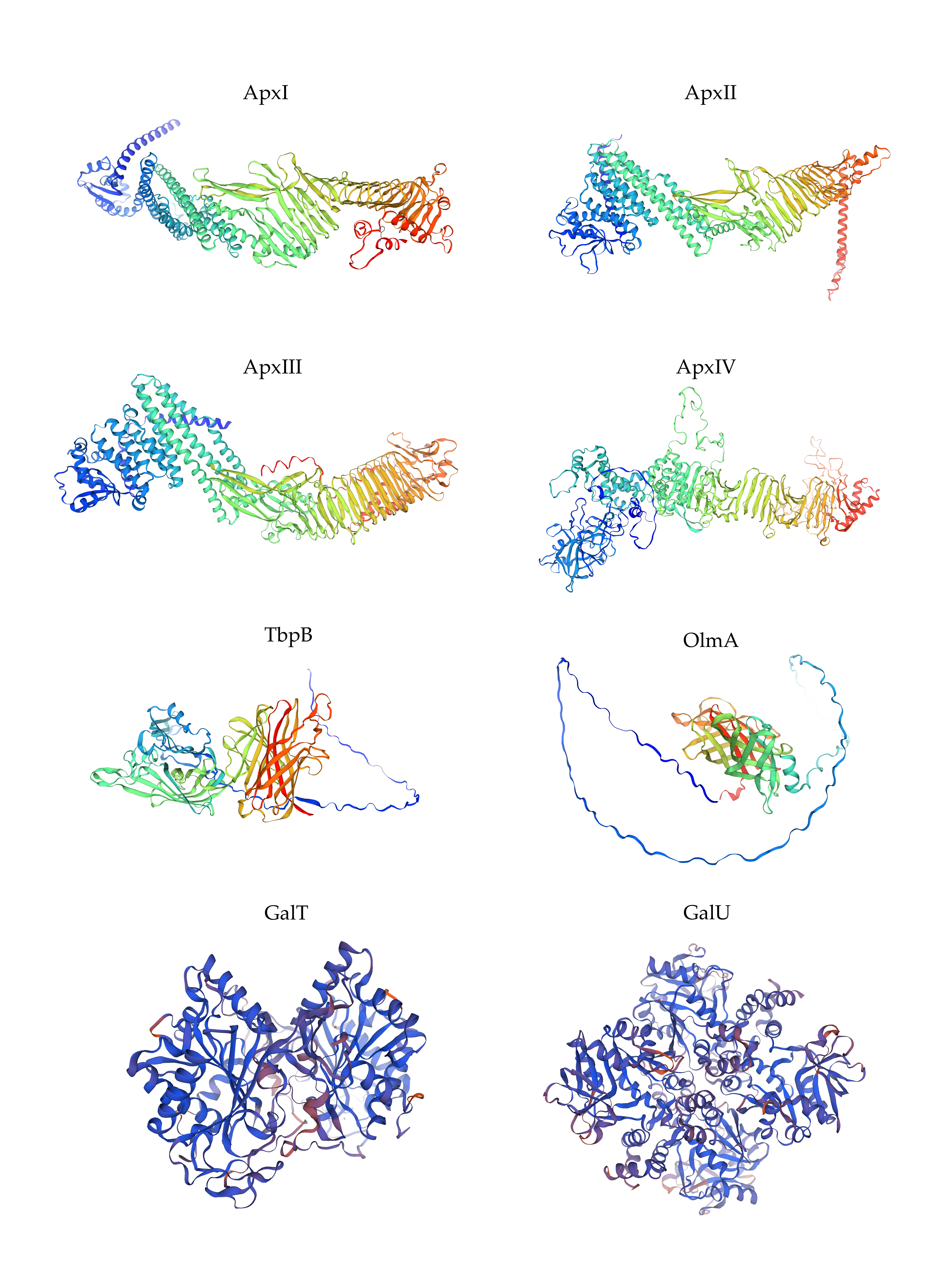

Supplement: Supplementary file 1 [file vetsci-12-00414-s001.zip › Supplementary File/Figure S5. The prediction results of the tertiary structures of the selected 8 proteins.tif]

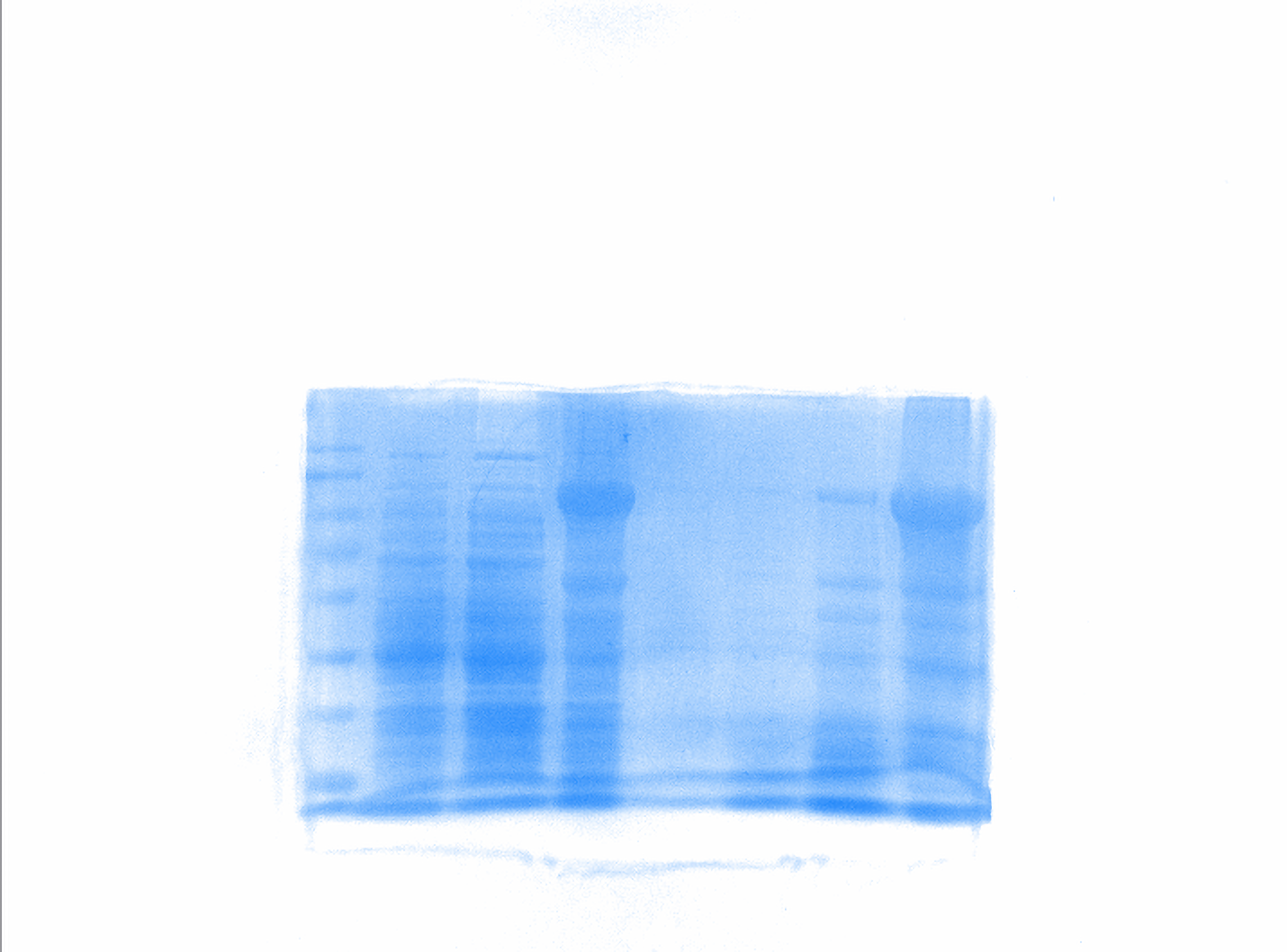

Supplement: Supplementary file 1 [file vetsci-12-00414-s001.zip › Supplementary File/Figure S6-S9. Original SDS-PAGE and Western Blotting images of ApxI and ApxII/Figure S6. Original SDS-PAGE image of ApxI.png]

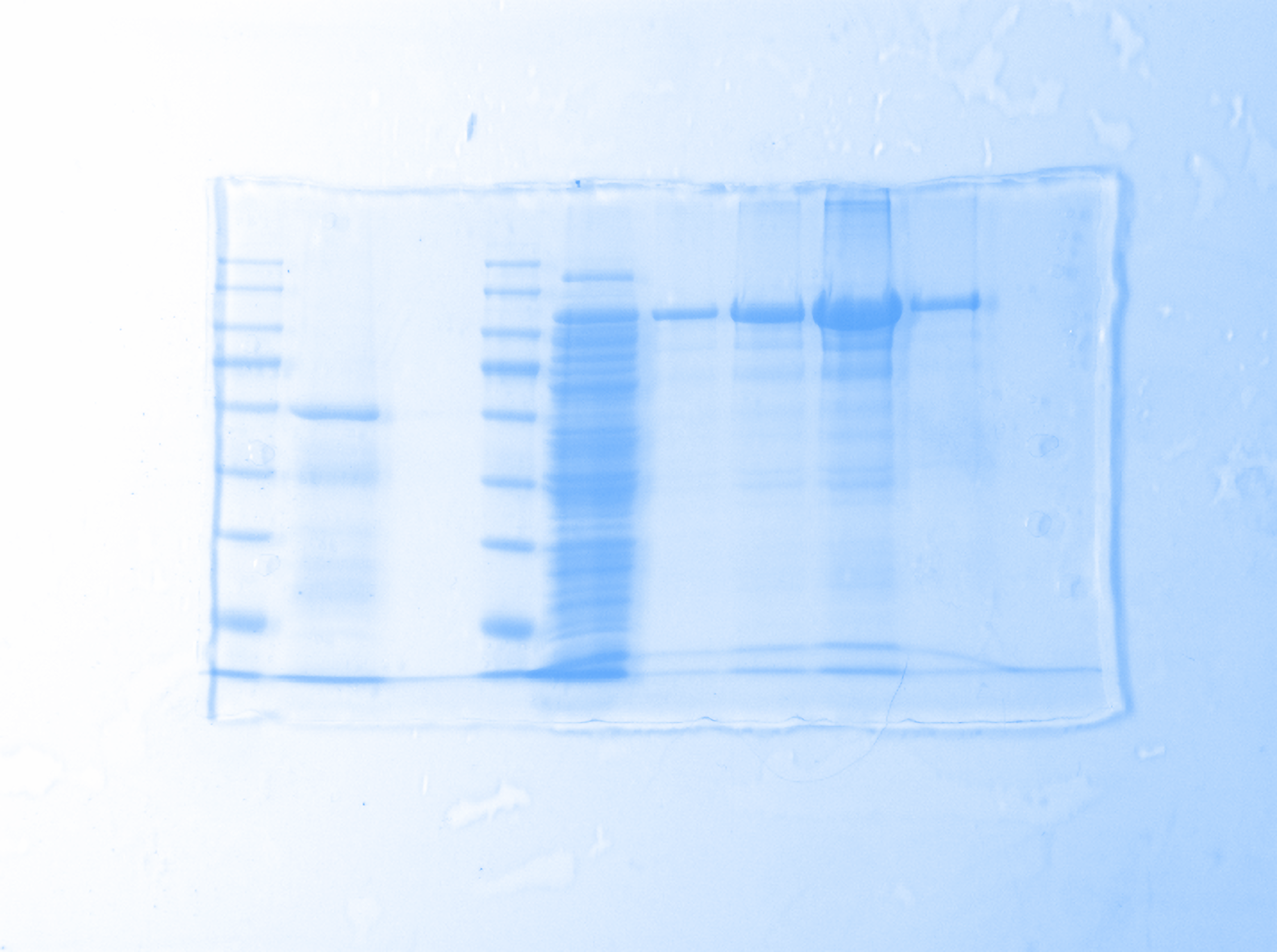

Supplement: Supplementary file 1 [file vetsci-12-00414-s001.zip › Supplementary File/Figure S6-S9. Original SDS-PAGE and Western Blotting images of ApxI and ApxII/Figure S7. Original SDS-PAGE image of ApxII.png]

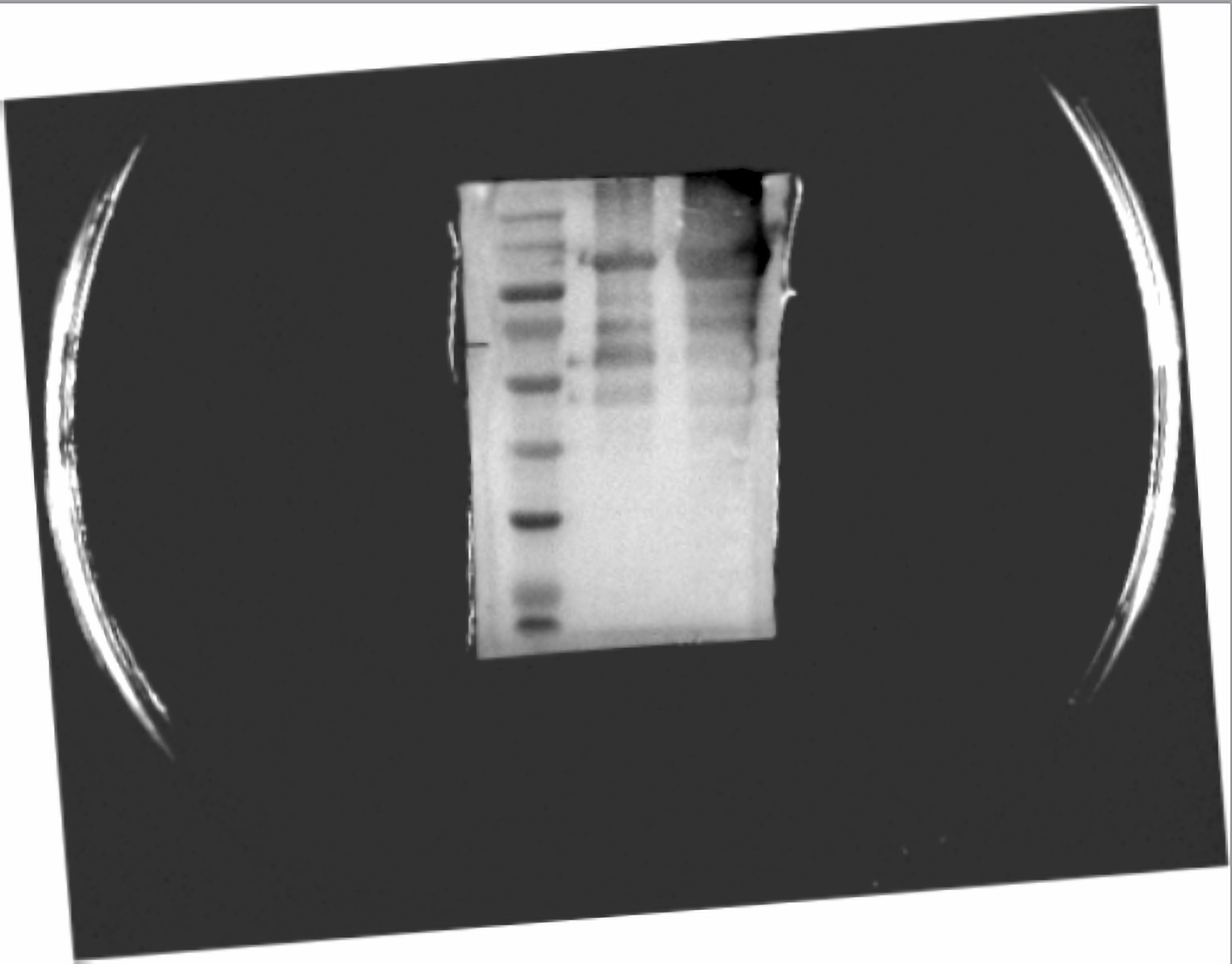

Supplement: Supplementary file 1 [file vetsci-12-00414-s001.zip › Supplementary File/Figure S6-S9. Original SDS-PAGE and Western Blotting images of ApxI and ApxII/Figure S8. Original Western Blotting image of ApxI(His-tag monoclonal antibody).png]

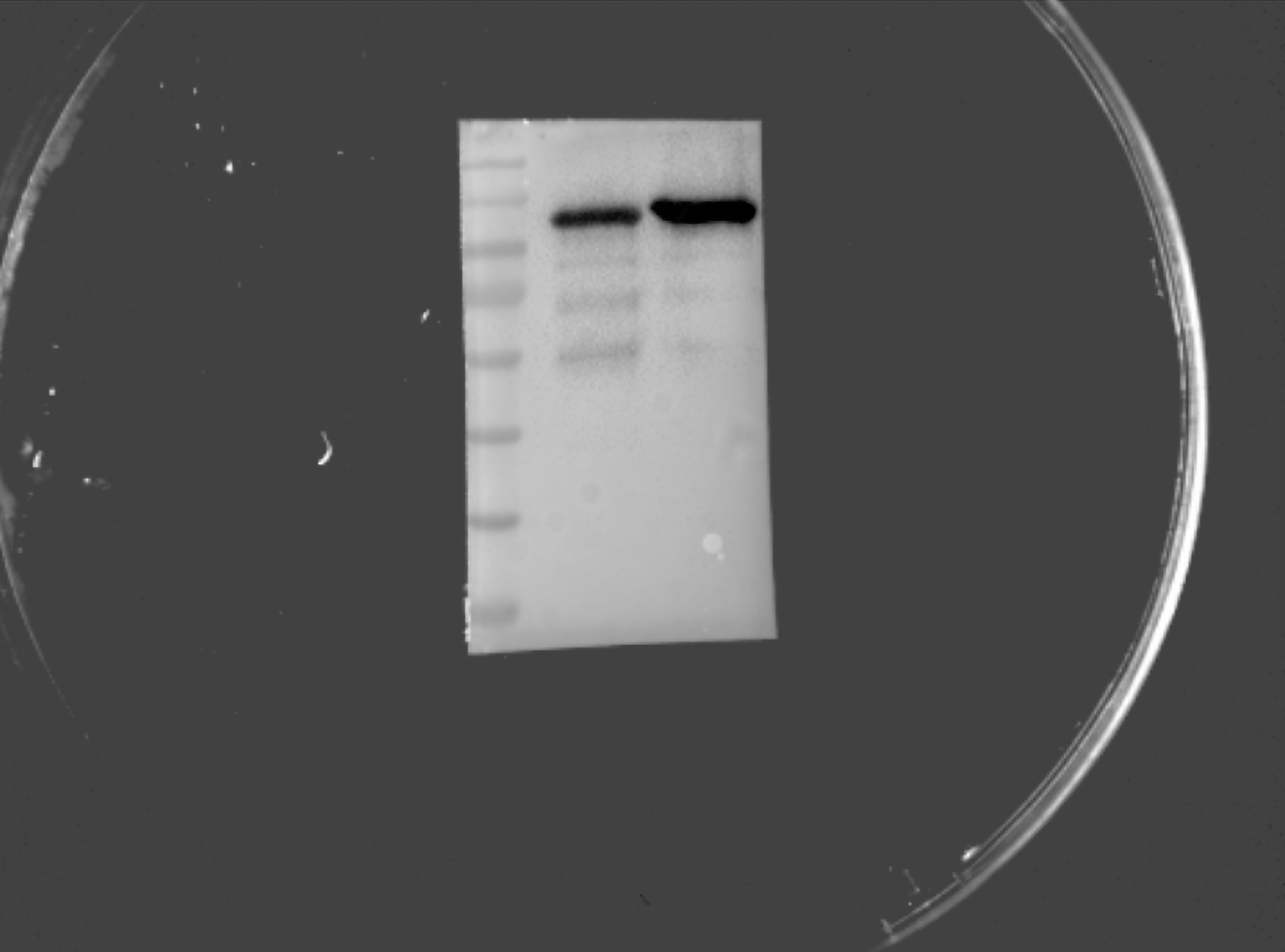

Supplement: Supplementary file 1 [file vetsci-12-00414-s001.zip › Supplementary File/Figure S6-S9. Original SDS-PAGE and Western Blotting images of ApxI and ApxII/Figure S9. Original Western Blotting image of ApxII(His-tag monoclonal antibody).png]
